# Compound-specific stable isotope analyses of fatty acids indicate feeding zones of zooplankton across the water column of a subalpine lake

**DOI:** 10.1007/s00442-024-05574-3

**Published:** 2024-06-03

**Authors:** Matthias Pilecky, Samuel K. Kämmer, Katharina Winter, Radka Ptacnikova, Travis B. Meador, Leonard I. Wassenaar, Patrick Fink, Martin J. Kainz

**Affiliations:** 1 WasserCluster Lunz - Biologische Station GmbH, Inter-University Center for Aquatic Ecosystem Research, Dr. Carl-Kupelwieser Promenade 5, 3293 Lunz/See, Austria; 2 Research Lab for Aquatic Ecosystem Research and Health, Donau-Universität Krems, Dr. Karl-Dorrek Straße 30, 3500 Krems, Austria; 3 University of Southern Bohemia, Na Sádkách 7, 370 05 České Budějovice, Czech Republic; 4 Biology Center CAS, Na Sádkách 7, 370 05 České Budějovice, Czech Republic; 5 Department River Ecology, Helmholtz Centre for Environmental Research, UFZ, Brückstraße 3a, 39114 Magdeburg, Germany; 6 Department Aquatic Ecosystem Analysis and Management, Helmholtz Centre for Environmental Research, UFZ, Brückstraße 3a, 39114 Magdeburg, Germany

**Keywords:** Trophic ecology, Zooplankton feeding, Compound-specific stable isotopes, Deuterium, Diel vertical migration, Essential fatty acids, GC-IRMS

## Abstract

Spatial and temporal zooplankton feeding dynamics across the water column of lakes are key for understanding site-specific acquisition of diet sources. During this 6-week lake study, we examined stable carbon (δ^13^C) and nitrogen (δ^15^N) isotopes and conducted compound-specific fatty acid (FA) stable isotope analysis (CSIA) of edible seston in the epi-, meta-, and hypolimnion, and zooplankton of Lake Lunz, Austria. We predicted that CSIA of essential FA can discern the foraging grounds of zooplankton more accurately than the commonly used bulk stable isotopes. The δ^13^C and δ^15^N values of seston from different lake strata were similar, whereas a dual CSIA approach using stable carbon and hydrogen isotopes of FA (δ^13^C_FA_ and δ^2^H_FA_) provided sufficient isotopic difference in essential FA to discern different lake strata-specific diet sources throughout the study period. We present a CSIA model that suggests strata-specific foraging grounds for different zooplankton groups, indicating higher preference of cladocerans for feeding on epilimnetic diet sources, while calanoid copepods retained more hypolimnetic resources. The CSIA approach thus yields strata-specific information on foraging strategies of different zooplankton taxa and provides more details on the spatial and temporal trophodynamics of planktonic food webs than commonly used bulk stable isotopes.

**Supplementary Information:**

The online version contains supplementary material available at 10.1007/s00442-024-05574-3.

## Introduction

Aquatic food webs are fueled by dietary compounds, including organic carbon, macronutrients, and essential biochemicals, which are trophically transferred from basal resources to subsequent consumers. Essential dietary compounds are mostly synthesized by primary producers (Twining et al. 2021), yet imbalances between the dietary availability and demand of essential biochemcials can be a limiting factor for somatic growth and reproduction of consumers, such as zooplankton (Müller-Navarra 2008). One of the most important groups of essential biochemicals in aquatic food webs are dietary omega-3 and omega-6 long-chained polyunsaturated fatty acids (n-3 and n-6 LC-PUFA). Zooplankton, as the case for other animals, cannot synthesize n-3 or n-6 PUFA de novo (Voss et al. 1991) and thus depend on the dietary provision of the essential n-3 and n-6 PUFA; i.e., α-linolenic acid (18:3 n-3, ALA) and linoleic acid (18:2 n-6, LIN), from their diet. Omega-3 LC-PUFA are particularly important for the development of neural systems (Pilecky et al. 2021) as well as for survival of zooplankton (Taipale et al. 2011; Titocci and Fink 2022) and fishes (Copeman et al. 2002), while n-6 LC-PUFA are important for immune regulation, coagulation, and osmoregulation (Castro et al. 2016). As an evolutionary response to the dietary requirement of n-3 and n-6 LC-PUFA, many herbivorous animals have evolved the capability to convert the essential n-3 and n-6 PUFA to the physiologically active LC-PUFA eicosapentaenoic acid (20:5 n-3, EPA) and docosahexaenoic acid (22:6 n-3, DHA), and arachidonic acid (20:4 n-6, ARA), respectively.

In lakes, dietary access to the most edible particle size for herbivorous zooplankton (‘seston’, primarily composed of microalgae < 30 µm; (Burns 1968; Vanderploeg and Paffenhöfer 1985) varies with depth and season (Rasconi et al. 2018). Lake zooplankton typically perform diel vertical migration (DVM) by moving to deeper, dark water layers during the daytime to minimize predation risk and moving to the algae-rich upper water layers at night to optimize feeding success (Ringelberg 2009). The diel vertical migration should thus result in low average feeding rates when zooplankton dwell in low resource environments, e.g., the hypolimnion. It is therefore reasonable to assume that the dietary acquisition of physiologically required LC-PUFA is highest in algae-rich water layers, provided that the highest biomass of LC-PUFA-rich algae, such as dinoflagellates, diatoms, chrysophytes, and cryptophytes (Lang et al. 2011; Taipale et al. 2013), is found at similar shares at algae-rich water layers.

Among zooplankton, cladocerans differ in their feeding behaviour from copepods in that they are mainly non-selective filter feeders, while most herbivorous copepods selectively graze on their edible prey (Titocci and Fink 2022). Moreover, cladocerans retain EPA as its main n-3 LC-PUFA and partially retro-convert DHA to EPA (Pilecky et al. 2022b; von Elert 2002), while copepods require high quantities of DHA for somatic growth and reproduction (Burns et al. 2010; Pilecky et al. 2022b). The different PUFA requirements for cladocerans and copepods raises the question where these zooplankton groups find their optimal feeding depths in lakes.

Understanding spatial and temporal zooplankton feeding dynamics throughout the lake water column requires high temporal sampling resolution and biochemical methods that indicate depth-specific zooplankton feeding grounds. Thus far, bulk-tissue stable isotope analyses have been frequently used to assess diet sources and feeding strategies of lake zooplankton (e.g., δ^13^C, δ^15^N; Harvey and Kitchell 2000; Morlock et al. 2017; Wilkinson et al. 2014). However, bulk-tissue stable isotope analyses of planktonic organisms often face two inherent problems; 1) in the typically used stable isotope mixing models, the number of potential diet sources is limited to the number of isotopes plus one (Parnell et al. 2013), and; 2) the stable isotope composition of potential diet sources must be sufficiently different from each other, which is often not the case even in large or deep lakes (Harvey and Kitchell 2000; Lee et al. 2013). The limitations of stable isotope analyses may be overcome using compound-specific stable isotope analyses (CSIA) of FA or amino acids (Young et al. 2015). In aquatic ecosystems, FA-CSIA have been used to investigate FA metabolism (De Troch et al. 2012; Pilecky et al. 2022b), to identify fish mobility in mountain streams (Pilecky et al. 2022d), to discern different functional feeding groups of zooplankton and benthic invertebrates (Kohlbach et al. 2023; Kürten et al. 2013), and to distinguish between aquatic and terrestrial essential FA (Twining et al. 2020) as well as among plankton containing the same FA (Burian et al. 2020; Twining et al. 2020). However, it has so far never been used to track the spatial (lake depth) and temporal variability of diet sources in zooplankton of deeper lakes.

We examined stable isotopes (δ^13^C and δ^15^N) and compound-specific stable isotopes of FA of the most edible plankton size (seston) and zooplankton of Lake Lunz, Austria, for six weeks, aiming at discerning feeding depths of the herbivorous crustacean zooplankton (*Daphnia longispina*, *Bosmina longirostris*, *Eudiaptomus gracilis*) and the omnivorous copepod *Cyclops abyssorum* of this subalpine lake. We sampled the phytoplankton in three different lake strata; (1) the epilimnion, which is the most upper layer (up to 5 m), with almost unchanged temperature and high light intensities, (2) the metalimnion, which is characterized by a sudden drop in temperature, but also the zone of the highest phytoplankton biomass, and (3) the hypolimnion, where oxygen concentration and phytoplankton biomass drop significantly and which is also characterized by stable temperatures at around 4 °C during stratification. Furthermore, we took integrated samples of zooplankton and split them at species level, *i.e.*, the herbivorous crustacean zooplankton (*Daphnia longispina*, *Bosmina longirostris*, *Eudiaptomus gracilis*) and the omnivorous copepod *Cyclops abyssorum*. A previous finding that the stable isotope values of the essential PUFA (LIN and ALA) in zooplankton of shallow fish ponds reflected those of diet sources (Pilecky et al. 2022b) provided impetus to test the hypothesis that CSIA of essential FA can discern the foraging grounds (lake strata) of zooplankton species with contrasting PUFA requirements more accurately than the commonly used stable carbon or nitrogen isotopes. As CSIA can also be linked to FA content, FA-specific stable isotopes have the potential to serve as a source-specific metric of diet quality across the water column, thus help elucidate the different strategies of zooplankton taxa to obtain their physiologically required FA.

## Methods

### Study site and sampling

Lake Lunz (68 ha; 47°51′ 10″ N, 15°3′ 10″ E, 608 m a.s.l., 34 m maximum depth) is a pristine subalpine, oligotrophic lake (McMeans et al. 2015). Several fish species are known to naturally occur in the lake, including Arctic charr (*Salvelinus alpinus*), Northern pike (*Esox lucius*), perch (*Perca fluviatilis*), brown trout (*Salmo trutta*), roach (*Rutilus rutilus*), and European minnow (*Phoxinus phoxinus*) (Kainz et al. 2017). Crustacean zooplankton is known to perform extensive DVM in Lake Lunz (Siebeck 1960). Water and air temperatures, as well as precipitation were routinely recorded (daily) at a nearby weather station. Automatic lake depth profile measurements (0.3 m resolution) are also routinely taken for water temperature, dissolved oxygen content, and chlorophyll-a three times a day using a Hydrolab^™^ HL7 multiparameter sonde (Ott HydroMet, Loveland, CO).

Lake seston and zooplankton samples were collected in the morning three times a week from a stationary platform above the deepest spot of the lake (33 m) from July 7 to August 11, 2021. Lake water was collected from three different strata, defined via the daily measured temperature profile: the epilimnion (2–3 m below the surface, less than 3 °C difference to surface temperature in the morning of the sampling day), metalimnion (8–10 m, depth determined by maximum change in temperature per depth; typically also the depth with the highest chlorophyll-α concentrations) and hypolimnion (25 m, zone of constant 4 °C throughout the day) using a Schindler trap (3.5 L; 5 times per strata). Seston (triplicates from 3 L) samples were filtered through a 30 µm mesh, retained on pre-combusted Whatman™ GF/C filters (0.7 µm), and stored at − 80 ºC. Subsamples (40 mL) were preserved (Lugol’s solution) for taxonomic composition analysis of phytoplankton using an inverted microscope (40 × magnification, Bresser, Rhede, Germany). Zooplankton were collected in triplicates by vertically hauling a plankton net (100 µm mesh size, 36 cm diameter) from 25 m depth to the surface. For evaluation of size dependent differences in foraging strategies, bulk zooplankton was separated on 500 µm and 250 µm mesh size filter cups, fully transferred into Falcon™ tubes (50 mL) by rinsing the filter with lake water. Bulk samples were frozen at − 80 °C, subsequently freeze-dried, separated to different species, and stored at − 80 °C until further analysis.

### Elemental and bulk stable isotope analyses

Freeze-dried seston filters were put into 4 × 9 mm tin capsules (IVA Analysetechnik, Meerbusch, Germany). Their stable-isotope (*δ*^13^C and *δ*^15^N) values were measured using a ThermoFisher Flash HT Plus™ Elemental Analyzer interfaced with a Conflo IV (Thermo Co., Bremen, Germany) to a continuous flow isotope-ratio mass spectrometer (Delta V Advantage, Thermo Co.). The sample δ-values were measured against reference gas injections of pure N_2_ and CO_2_ (Messer, Krefeld, Germany) and normalized to the AIR and PDB scales using international standards IAEA-N-1 and IAEA-N-2 for δ^15^N, and USGS24 and IAEA-CH-7 for δ^13^C, respectively.

### Gas chromatography and isotope-ratio mass spectrometry (GC-IRMS)

Lipids were extracted according to standard procedures described in detail elsewhere (Pilecky et al. 2023b). In brief, freeze-dried filters and zooplankton were homogenized and mixed with chloroform:methanol (2:1 vol/vol) following sonication, vortexing and centrifuging 3 times to remove nonlipid materials. Excess solvent was evaporated to a final volume of 1.5 mL under N_2_. Lipid content was quantified gravimetrically by transferring 2 × 10% of the extract into pre-weighted tin cups. For fatty acid methyl esters (FAME) formation, samples were incubated with sulfuric acid:methanol (1:100 vol/vol) for 16 h at 50 °C, following addition of KHCO_3_ and hexane. Samples were shaken, vortexed and centrifuged and the upper organic layers collected twice, pooled and concentrated under N_2_.

FAME were analyzed using a gas chromatograph (TRACE GC, ThermoFisher Scientific) equipped with an FID and a SUPELCO^™^ SP-2560 column (100 m, 25 mm i.d., 0.2 µm film thickness). Chromeleon 7™ was used for peak quantification. FAME were identified by comparison of their retention times with known standards (37-component FAME mix, 47,885-U, Supelco; Sigma-Aldrich, Bellefonte, Pennsylvania). Fatty acid concentrations were determined using external calibration curves based on known standard concentrations.

Compound-specific stable isotope analyses of FA (δ^13^C_FA_ and δ^2^H_FA_) were performed using a ThermoFisher Trace 1310 GC (ThermoFisher Scientific, Waltham, MA), connected via a ConFlo IV (Thermo Co.) to an isotope-ratio mass spectrometer (DELTA V Advantage, ThermoFisher) as described elsewhere (Pilecky et al. 2021). The FAME were separated using a VF-WAXms 60 m column, 0.25 mm ID, film thickness 0.25 µm or a VF-WAXms 30 m column, 0.32 mm ID, film thickness 1 µm (both Agilent, Santa Clara, CA). For *δ*^13^C analysis, analytes were oxidized to CO_2_ in a combustion reactor, filled with Ni, Pt and Cu wires, at a temperature of 1000 °C. For *δ*^2^H analysis, analytes were reduced to H_2_ by passing through a ceramic high-temperature reactor at 1200 °C. The temperature program for the 60 m GC column started at 80 °C, which was kept for 2 min, after which the temperature was raised by 30 °C min^−1^ to 175 °C, by 5 °C min^−1^ to 200 °C and finally by 2.4 °C min^−1^ to 250 °C, which was maintained for an additional 30 min. The temperature program for the 30 m GC column started at 80 °C, was help for 2 min, after which the temperature was raised by 30 °C min^−1^ to 175 °C, and then by 5 °C min^−1^ to 240 °C, which was held for 35 min.

The sample injection volumes were adjusted to obtain IRMS peak amplitudes between 300–8000 mV for ^12^CO_2_ and 1000–10000 mV for ^1^H_2_ for all peaks of interest, which was within the linear range of the IRMS system. For *δ*^2^H measurements, a H_3_^+^-factor determination was performed before and after each measurement sequence using a dilution series of the reference gas. The samples were run with consensus FAME-C20 standards (USGS70: *δ*^13^C =  − 30.53* ‰*, *δ*^2^H =  − 183.9* ‰*, USGS71: *δ*^13^C =  − 10.5* ‰*, *δ*^2^H =  − 4.9* ‰* and USGS72: *δ*^13^C =  − 1.54* ‰*, *δ*^2^H =  + 348.3* ‰*), which were used for drift and linear correction. The *δ*^13^C and *δ*^2^H value of individual FAME were determined by automated integration, defining 0.5 mV/s as start and end point of a peak and using individual background values. All peaks were validated and corrected manually if necessary. Fatty acid *δ*^13^C/*δ*^2^H values ($\delta {I}_{FA})$ were corrected for the methyl group addition during methylation according to the formula

$$\delta {I}_{FA}=(\left(n+1\right) \times \delta {I}_{FAME}-\delta {I}_{MeOH})/n$$

where $\delta {I}_{FAME}$ are the *δ*^2^H or *δ*^13^C values of the measured FAME and $\delta {I}_{MeOH}$ the *δ*^2^H or *δ*^13^C values of the methanol used during methylation and $n$ equals the total number of H-/C-atoms of each of the identified FAME molecules. Values for *δ*^13^C were referenced to the Vienna PeeDee Belemite (PDB) standard (^13^C:^12^C = 0.01118)

$$\delta {}_{{}}^{13} C_{FA} = \left( {\frac{{{}_{{}}^{13} C/{}_{{}}^{12} C_{Sample} }}{{{}_{{}}^{13} C/{}_{{}}^{12} C_{VPDB} }} - 1} \right) \times 1000$$

Values for *δ*^2^H were referenced against the Vienna Standard Mean Ocean Water (VSMOW) standard (^2^H:^1^H = 155.76 ppm)

$$\delta {}_{{}}^{2} H_{FA} = \left( {\frac{{{}_{{}}^{2} H/{}_{{}}^{1} H_{Sample} }}{{{}_{{}}^{2} H/{}_{{}}^{1} H_{VSMOW} }} - 1} \right) \times 1000$$

### Data analysis

Data analysis and graphics were performed in R (Version 4.0.2) using the packages *rstatix*, *ggplot2*, *ggpubr*, *lme4*, *vegan*, and *corrplot*. Data were tested for normality using Shapiro-Wilks test. Bayesian isotopic mixing models were generated in R, using the package *simmR*. Such models are commonly established for a two isotope (bulk δ^13^C and δ^15^N) approach (Parnell et al. 2013). We here extended this approach to two isotopes of two FA (δ^2^H and δ^13^C values of LIN and ALA) as source markers. Consumer-diet isotope discrimination factors used in the models were for δ^2^H_ALA_ − 12.4 ± 3.8 ‰, for δ^2^H_LIN_ − 22.2 ± 5.8 ‰, for δ^13^C_ALA_ − 0.05 ‰ ± 1.32, and for δ^13^C_LIN_ − 1.55 ‰ ± 1.2 based on experimental and field stable isotope data (Pilecky et al. 2022b). All values are presented as the mean ± standard deviation. All mass fractions are referred to dry weight.

## Results

### Physicochemical parameters, plankton biomass, and bulk stable isotopes

**Fig. 1:**
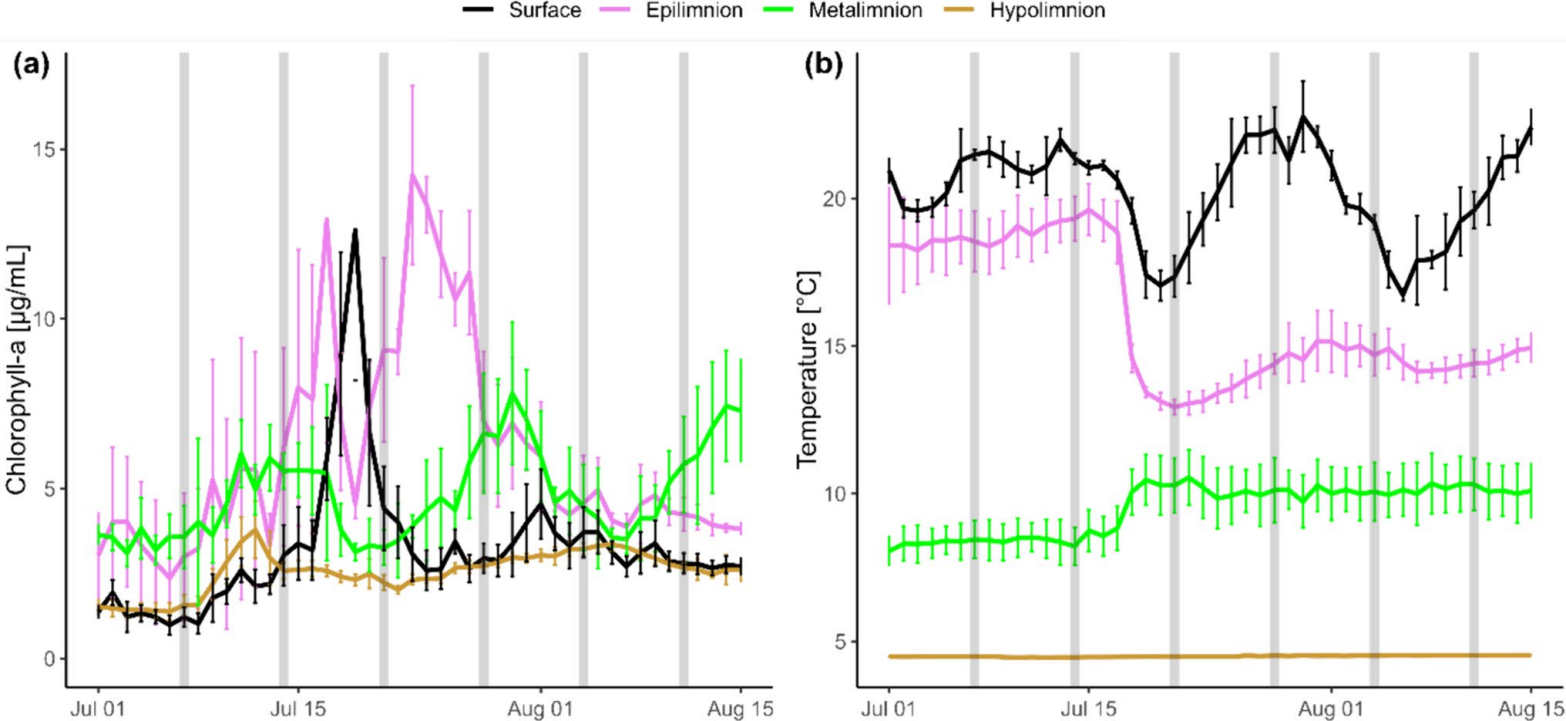
**a** Water temperature profiles and (**b**) chlorophyll-a content of epilimnion (2–3 m), metalimnion (6–10 m) and hypolimnion (25 m) of Lake Lunz over the study period. Gray bars indicate sampling time points of zooplankton genera

**Fig. 2:**
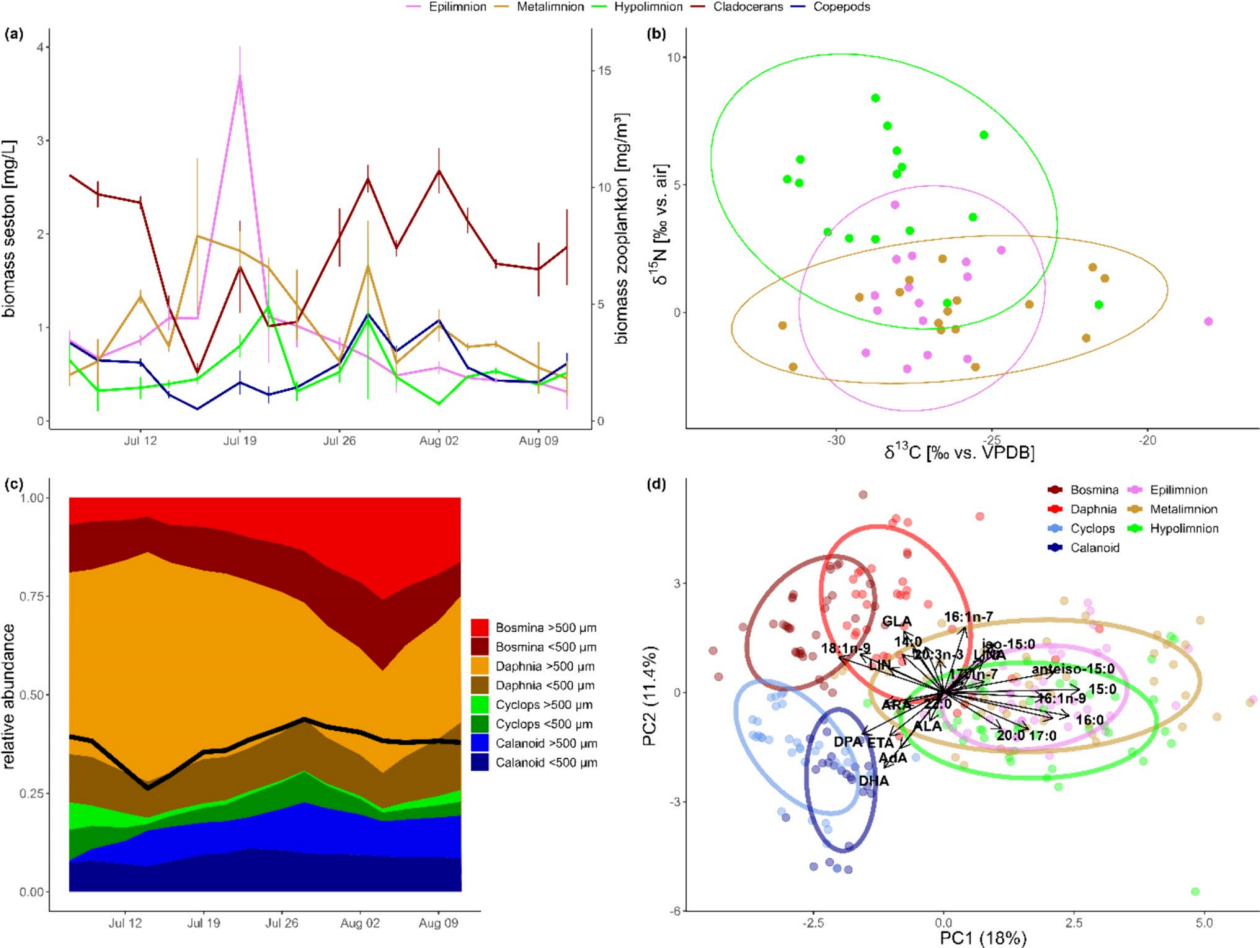
**a** Changes in biomass of seston (N = 3) and zooplankton (N = 6) over the study period. **b** Bulk stable-isotope biplots show a large overlap between seston samples of the different lake layers. **c** Relative abundance of zooplankton species during the study period; black line indicates the total ratio of small (< 500 µm) vs. large zooplankton. **d** Principal component analysis of fatty acid (%) of zooplankton and seston. Copepods were particularly enriched in LC-PUFA and differed in their fatty acid profiles from cladocerans

During the study period, an extreme precipitation event (> 100 mm / 24 h) occurred on July 17, 2021. This flooding event caused a large water temperature to drop in the upper lake layer by ~ 5 °C and a slight water temperature rise in the metalimnion (Fig. 1a). After the flooding event, chlorophyll-*a* concentrations peaked at the lake surface water on July 19 (12.6 µg/L ± 0.5), on July 23 in the epilimnion (14.2 µg/L ± 0.6), and on July 30 in the metalimnion (7.8 µg/L ± 0.1), whereas no chlorophyll-*a* peaks were observed in the hypolimnion (Fig. 1b). The high discharge transferred particulate matter to the lake, causing a high increase in turbidity with a reduction in Secchi depth from ~ 5 m to 0.7 m within 24 h, followed by a re-establishment of higher lake water transparency with a Secchi depth of 5 m over the next 10 days. Seston biomass peaked in the epilimnion on July 19 (3.7 mg/L ± 0.3) and in the hypolimnion on July 21 (1.2 mg/L ± 0.2), while a broader peak in seston biomass was observed in the metalimnion over the entire study period. Conversely, zooplankton biomass dropped from ~ 8 mg/m^3^ and ~ 4 mg/m^3^ for cladocerans and copepods, respectively, to approximately half during the flooding event (Fig. 2a). Throughout the study period, cladocerans (*Daphnia longispina* and *Bosmina longirostris*) were more abundant than copepods, whereby calanoids (*Eudiaptomus gracilis*) were more abundant than cyclopoids (*Cyclops abyssorum*) (Fig. 2c).

At the beginning of the study period (prior to the extreme rain event), seston in the epi- and metalimnion consisted mainly of *Dinobryon, Gymnodium*, and *Cyclotella,* and traces of *Asterionella* in the metalimnion. In the hypolimnion, seston consisted mostly of detritus with traces of *Dinobryon* and diatoms. Following the flooding event, all lake water samples were dominated by organic debris that appeared to be disassembled chrysophytes and traces of diatoms. By the end of the study period, *Dinobryon, Gymnodinium, Asterionella, Mallomonas* and *Coleps spp.* were detected in the epi- and metalimnion, and *Ceratium* and *Cryptomonas spp.* in the metalimnion. The hypolimnion contained only organic and particulate detritus (see suppl. information).

Bulk stable isotope data of seston of all layers were generally enriched in ^13^C (pooled data; n = 144: − 28.5 ‰ ± 3.6) compared to those of zooplankton (n = 96; − 31.9 ‰ ± 1.0, t-test, p < 0.001). In zooplankton, bulk δ^13^C values were similar between cladocerans and copepods (each n = 48; − 31.9 ‰ ± 1.0 vs. − 32.9 ‰ ± 1.5, Tukey, p = 0.30), as was the case for seston of the meta- and epilimnion (each n = 48; − 26.3 ‰ ± 3.1 vs. − 26.7 ‰ ± 2.6, Tukey, p = 0.92). However, the δ^13^C values of cladocerans and copepods differed slightly from those of hypolimnetic seston (− 28.1 ‰ ± 2.6; Tukey, vs. epilimnion: p = 0.036, vs. metalimnion: p = 0.003). Apart from a temporally short enrichment in δ^15^N values in seston of the hypolimnion at the beginning of the study, no significant difference in δ^15^N values of seston of the different lake layers was observed (Fig. 2b).

### Fatty acid composition of seston and zooplankton

Zooplankton were more than twice as lipid rich (*Bosmina*: 304 mg/g ± 81; *Daphnia*: 273 mg/g ± 100; *Cyclops*: 295 mg/g ± 119; *Eudiaptomus*: 218 mg/g ± 53) as seston (epilimnion: 109 mg/g ± 53; metalimnion: 85 mg/g ± 53; hypolimnion: 111 mg/g ± 62). The mean content of individual FA in seston of the different layers was not significantly different, except for DHA and LIN, which both had higher mass fractions (ANOVA, Tukey, p < 0.01) in seston of the hypolimnion compared to the metalimnion and epilimnion (DHA: epilimnion: 0.9 mg/g ± 0.3, metalimnion: 1.2 mg/g ± 0.6, hypolimnion: 2.0 mg/g ± 1.1; LIN: epilimnion: 0.7 mg/g ± 0.3, metalimnion: 1.0 mg/g ± 0.6, hypolimnion: 1.5 mg/g ± 1.0).

In contrast to seston of the different lake layers, all zooplankton genera differed in their FA profiles (MANOVA, Pillai post-hoc, p < 0.001, Fig. 2d). Most notably, higher DHA contents were found in *Eudiaptomus* (21.4 mg/g ± 12.9) and *Cyclops* (21.6 ± 8.2) compared to *Bosmina* (6.9 mg/g ± 3.1, Tukey, p < 0.001), which in turn had higher DHA contents than *Daphnia* (2.2 mg/g ± 1.5, Tukey, p = 0.026). In contrast, EPA only differed between *Cyclops* and *Bosmina* (7.4 mg/g ± 4.6 vs. 14.2 mg/g ± 12.1, Tukey, p = 0.004; *Eudiaptomus* and *Daphnia* both 9.4 mg/g ± 7.1). No differences were found among ALA contents of zooplankton. The LIN contents were both higher (Tukey, p < 0.05) in *Bosmina* (11.1 mg/g ± 8.7) and *Cyclops* (10.3 mg/g ± 7.9) than in *Daphnia* (6.2 mg/g ± 4.8) and *Eudiaptomus* (5.6 mg/g ± 5.5). The n-3/n-6 PUFA ratio was lower in *Daphnia* compared to copepods (2.3 ± 1.3 vs. 4.8 ± 3.0, respectively).

### High variation of fatty acid composition and stable isotopes in seston

**Fig. 3:**
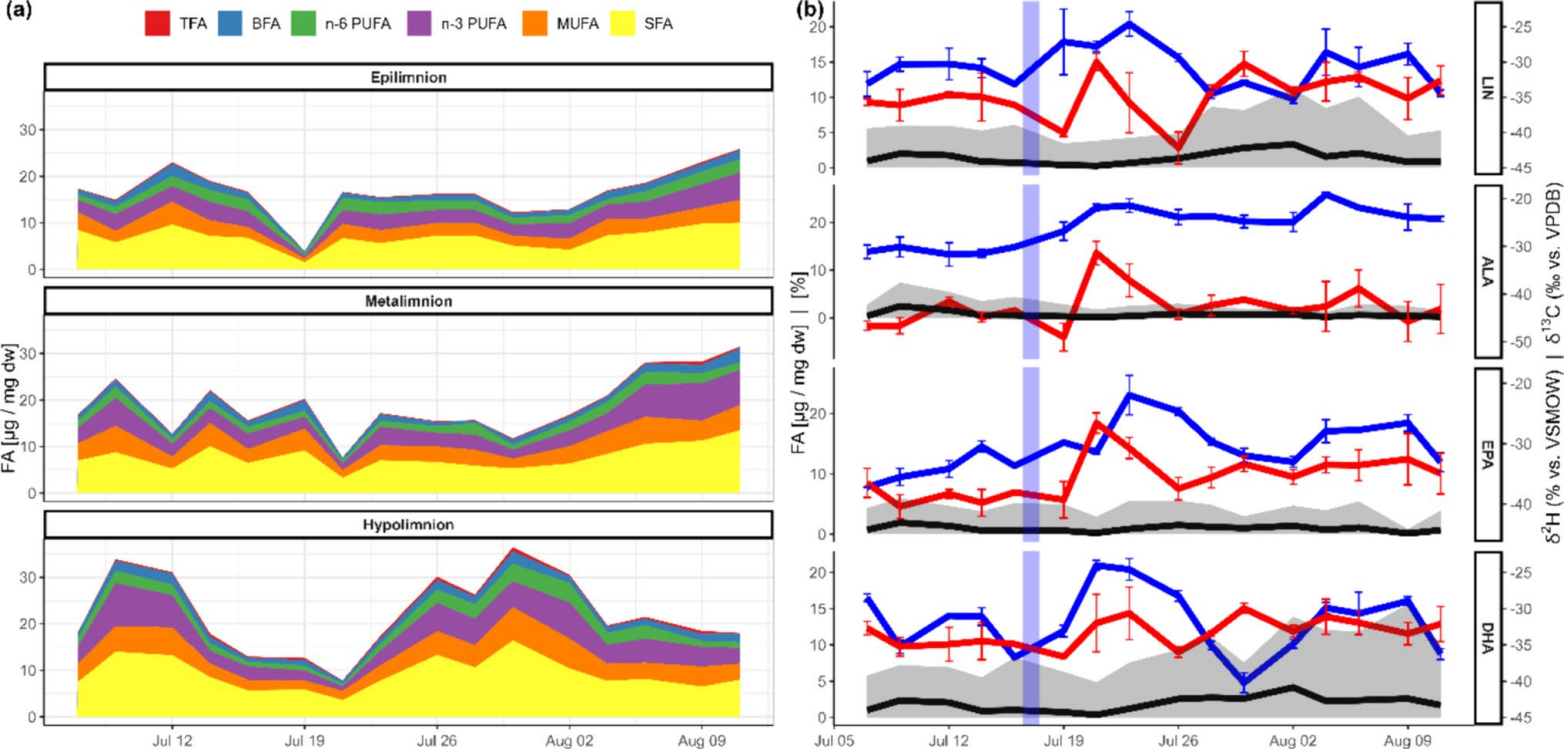
Time dependent changes in seston FA mass fraction and isotopic composition during the study (N = 3). **a** mass fractions of different FA classes in the seston samples in the hypolimnion (blue line indicates the extreme event); **b** PUFA of seston of the hypolimnion as an example for changes in fatty acid content and isotopic composition during the study period (N = 3). Changes in FA mass fraction (black line), relative FA mass fraction compared to total FA (gray area), δ^2^H (blue, in % vs. VSMOW) and δ^13^C (red in ‰ vs. VPDB) following the precipitation event. Changes in other layers and of other FA can be seen in Figure S1 and Table S2

Mass fractions of all FA groups in seston decreased significantly immediately after the extreme precipitation event in the epilimnion and two days later in the metalimnion. In the hypolimnion, a three-fold increase in mass fractions of all FA groups was observed a week before and after the precipitation event (Fig. 3a). While the relative content of individual FA did not change during the first general increase of FA in hypolimnion, the relative content of LIN_Hyp_ and DHA_Hyp_ increased from 4.0% ± 0.4 to 9.6% ± 1.3 and 6.6% ± 1.4 to 12.3% ± 1.2, respectively, during the second raise. This increase was preceded by an increase in δ^2^H_LIN_ (− 260 ‰ ± 20; before: − 315* ‰* ± 15) and δ^2^H_DHA_ (− 266 ‰ ± 28; before: − 325 ‰ ± 34), together with seston biomass increase, however without changes in δ^13^C values. The mass fractions of ALA_Hyp_ decreased after the flooding event, from 1.1 µg/mg ± 0.9 to 0.3 µg/mg ± 0.1 and 4.7% ± 1.8 to 2.2% ± 0.7, respectively, and remained at this level until the end of the study period. In parallel to the change in ALA content, δ^2^H_ALA_ values increased (before: − 309 ‰ ± 7; after: − 236 ‰ ± 29) and δ^13^C_ALA_ (before: − 44.6 ‰ ± 2.2; after: − 37.6 ‰ ± 1.2) (Fig. 3b).

In parallel to changes in FA mass fractions, changes in δ^2^H and δ^13^C values were observed. The δ^2^H_ALA_ values showed the largest differences among the lake water layers of all FA before the precipitation event (epilimnion: − 207 ‰ ± 22; metalimnion: − 232 ‰ ± 15; hypolimnion: − 309 ‰ ± 7, ANOVA, F_2,40´_ = 118.5, p < 0.001, Tukey all-comparisons p < 0.001), but similar thereafter (epilimnion: − 238 ‰ ± 16; metalimnion: − 226 ‰ ± 17; hypolimnion: − 236 ‰ ± 29, ANOVA, F_2,42_ = 0.277, p = 0.759). Before the event, δ^2^H_LIN_ values were significantly different (Tukey, p < 0.01) between the epilimnion and the other lake layers, but not between the meta- and hypolimnion; after the precipitation event, the δ^2^H_LIN_ values of the hypolimnion differed from those of the meta- and epilimnion. Approximately two weeks after the precipitation event, the δ^2^H_LIN_ values returned to their pre-event levels. The δ^13^C_LIN_ and δ^13^C_ALA_ values of seston were different among the individual lake layers before the flooding event, but the δ^13^C_ALA_ differed between the meta- and hypolimnion immediately after the flooding event. The δ-values and plots for FA of all layers before and after the flooding event are shown in the supplemental materials (Fig S1 and Table S2).

### Fatty acid stable isotopes of zooplankton

In contrast to seston samples, zooplankton showed almost no variation in the FA mass fractions and stable isotope values throughout the study period, despite the precipitation event. Three weeks after the event, zooplankton δ^2^H and δ^13^C values diverted significantly in ALA and LIN from the period before (MANOVA, Pillai, p < 0.05) for *Daphnia*, *Bosmina* and cyclopoids, but not calanoids, which remained at similar isotopic values throughout the study.

**Fig. 4:**
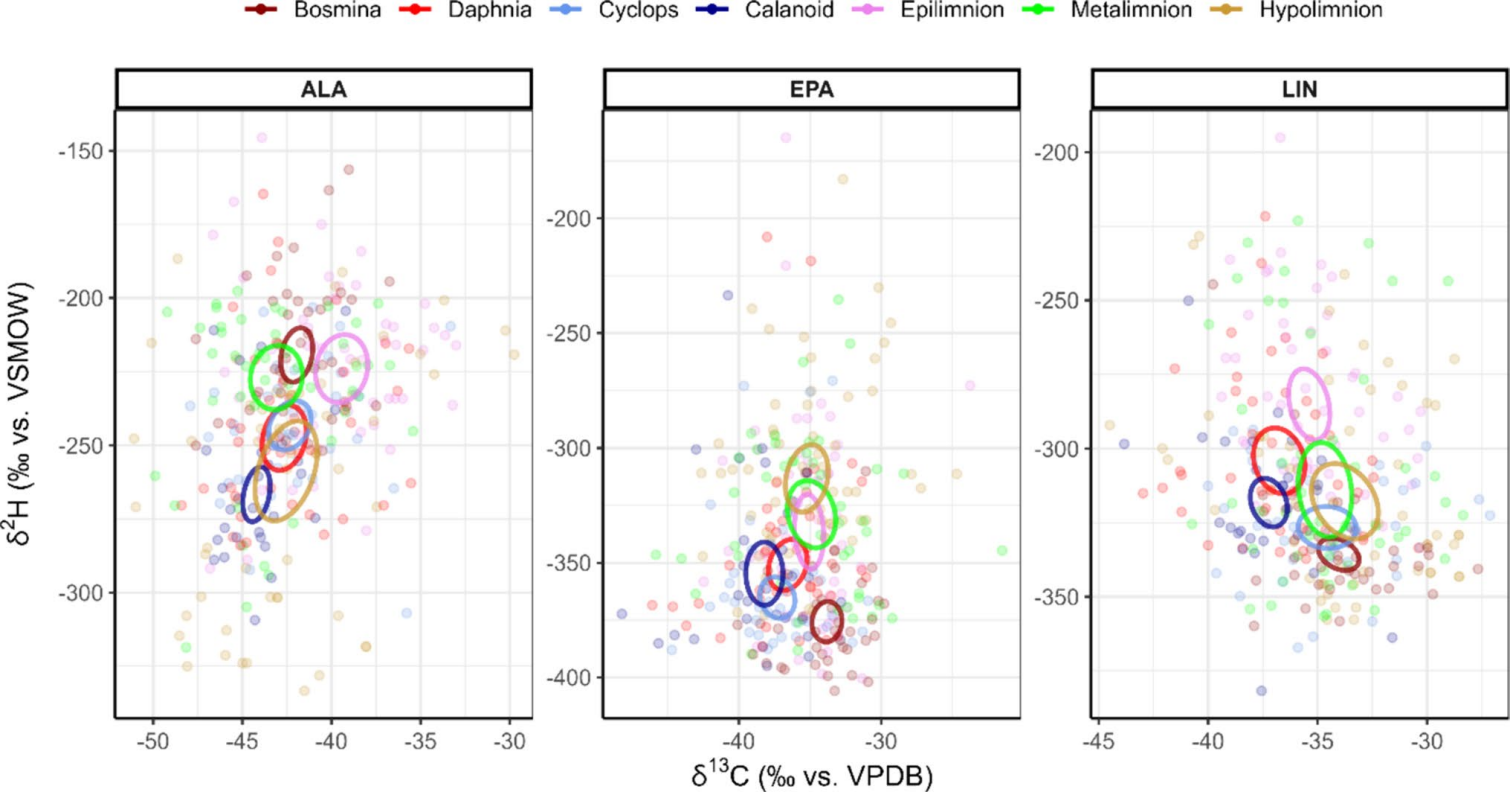
Isotopic biplot of δ^2^H and δ^13^C values of polyunsaturated fatty acids. LIN and ALA cannot be synthesized de novo by consumers and thus have to be acquired via diet. Thus, apart from a small trophic fractionation factor, they directly reflect the dietary sources of a consumer. On the other hand, EPA can be bioconverted from precursors, or, if not physiologically required, used as energy sources by consumers, thus altering the isotopic signature. For example, δ^2^H_EPA_ values of consumers tend to be lower than δ^2^H_EPA_ values of the potential dietary sources, which is most likely due to endogenous bioconversion from ALA (see Pilecky et al. 2022a, b, c, d)

Dual carbon and hydrogen analysis revealed differences in stable isotopes of ALA and LIN (MANOVA, Pillai, p < 0.05) between the four zooplankton genera, except for LIN between *Daphnia* and *Cyclops* and ALA between *Cyclops* and *Bosmina*, and between *Daphnia* and calanoids. Cladocerans had higher mean δ^2^H_ALA_ values (*Bosmina*: − 219.9 ‰ ± 24.6; *Daphnia*: − 239.5 ‰ ± 28.2) than copepods (cyclopoids: − 245.3 ‰ ± 21.2; calanoids: − 260.0 ‰ ± 26.3), while δ^13^C_ALA_ values were on average only ~ 2 ‰ lower in calanoids (− 44.1 ‰ ± 2.0) than in the other genera. The mean δ^2^H_LIN_ values of cladocerans were isotopically lighter (− 330.5 ‰ ± 19.3 in *Bosmina* and − 297.5 ‰ ± 27.5 in *Daphnia*) than those of δ^2^H_ALA_, while only a narrow isotopic range in δ^13^C_LIN_ values was observed (− 37.3 ‰ ± 1.8 in calanoids and − 34.1 ‰ ± 2.3 in *Bosmina*). The pooled δ^2^H and δ^13^C values of LIN and ALA matched between seston and zooplankton, whereas δ^2^H_EPA_ values were lower in zooplankton than in seston (− 394.7 ‰ ± 18.9 vs. − 337.1 ‰ ± 41.9, Tukey, p < 0.001), but no difference was found in δ^13^C_EPA_ values between seston and zooplankton (Fig. 4, Table S3). Dual-carbon and hydrogen isotope biplots of FA between zooplankton with different body-size (> 500 µm and < 500 µm) only revealed significant differences in *Bosmina* for LIN (Pillai, p = 0.015) and SDA (Pillai, p = 0.033).

### Bayesian diet source modelling

**Fig. 5:**
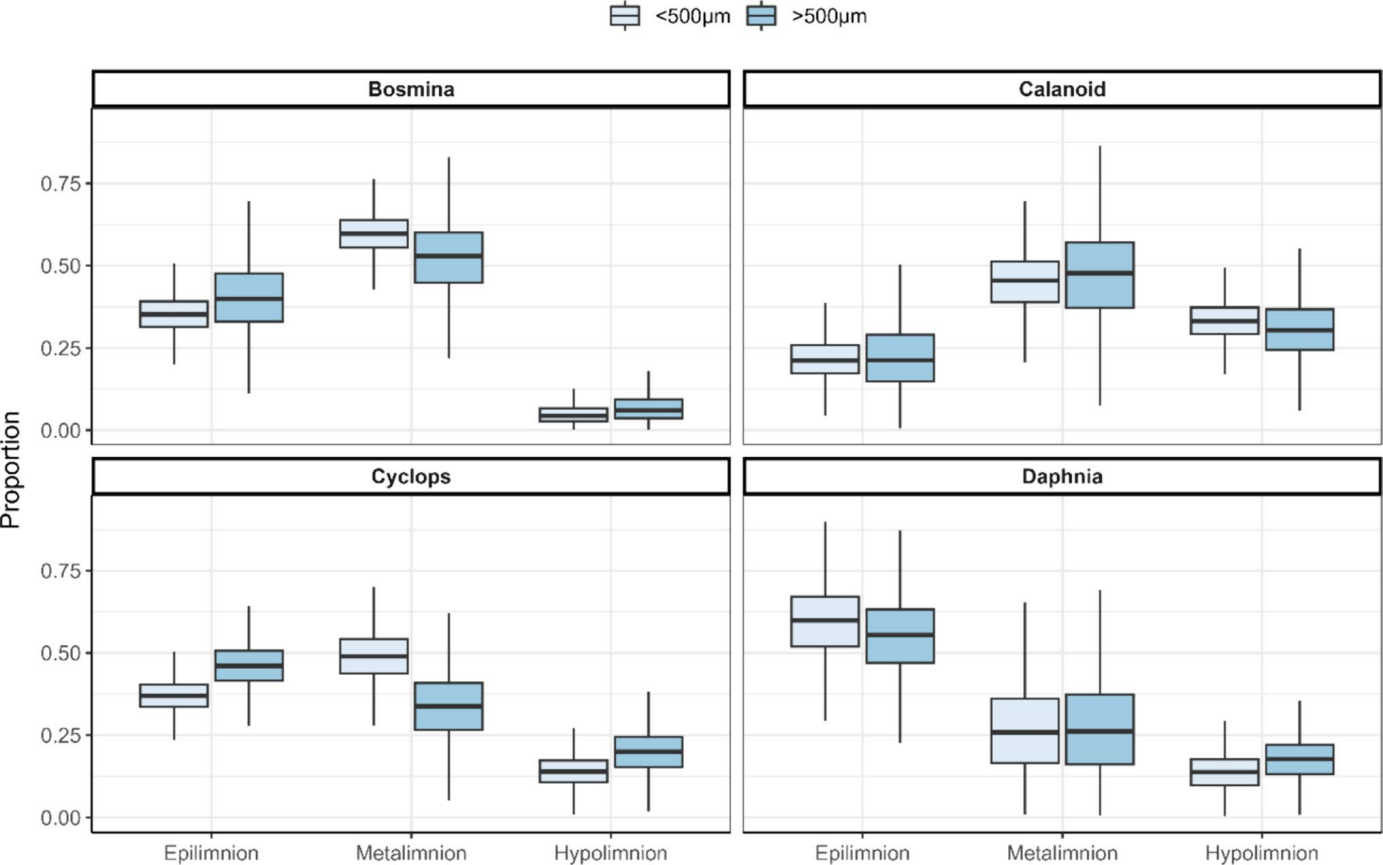
Bayesian mixed models using δ^2^H and δ^13^C values of LIN and ALA indicate preferential foraging layers of the zooplankton genera. Notably, a high preference of *Daphnia spp*. for the epilimnion was found, while calanoid copepods preferred deeper layers. The model suggests a slight tendency for deeper layers for larger size Daphnia and calanoids compared to their smaller conspecifics

A Bayesian stable isotope mixing model was performed using the stable isotope values of the essential FA LIN and ALA. These cannot be synthesized de novo by zooplankton. Therefore, their presence in consumer tissue indicates assimilation from the respective diet. *Daphnia* had the highest probability of feeding on epilimnetic seston (0.59 ± 0.13) followed by *Cyclops* (0.37 ± 0.05), *Bosmina* (0.33 ± 0.05) and calanoids (0.16 ± 0.05). Conversely, calanoids were the only zooplankton group with significant diet contributions attributed to hypolimnetic seston (0.32 ± 0.06). While the model suggested a higher preference of larger-sized *Daphnia* and *Eudiaptomus* for feeding on metalimnetic seston, *Cyclops* feeding was more pronounced in the epilimnion. No difference in feeding preferences was observed between the two *Bosmina* size classes (Fig. 5).

## Discussion

As hypothesized, δ^13^C and δ^15^N values were unable to discriminate among zooplankton diet sources within the water layers of Lake Lunz, whereas compound-specific stable isotopes of FA clearly differed in δ^2^H and δ^13^C values among seston of the different strata (i.e., epi-, meta-, and hypolimnion). These differences included the essential PUFA LIN and ALA, which cannot be synthesized by consumers, and must have thus originated from the diet at specific lake depths. The isotopic values for LIN and ALA of consumers, corrected for trophic fractionation, reflected the range of values found in the diet, thereby tracking the feeding grounds within the water column of different zooplankton species. Zooplankton foraging behavior, as estimated by the Bayesian isotope mixing model, was similar to earlier extensive DVM studies in the same lake with the same zooplankton focal taxa (Siebeck 1960). *Daphnia longispina* was found to be associated with epilimnetic resources, while *C. abyssorum* and *B. longirostris* showed higher dietary dependency on metalimnetic seston. *Cyclops abyssorum* is considered omnivorous, therefore FA might have been transferred via intermediate trophic links; e.g., via ciliates (Wickham 1995). While an additional trophic step might slightly increase the uncertainty due to potentially higher trophic isotope fractionation from seston to cyclops, mainly regarding δ^2^H values, it is still expected to be much smaller than the observed differences between the seston of the different layers. Furthermore, the high presence of *C. abyssorum* in the metalimnion has been observed several times in this lake (Siebeck 1960). *Eudiaptomus gracilis* was the only species to selectively retain hypolimnetic food sources, benefiting from PUFA-rich algal detritus that settled into the hypolimnion after of a massive precipitation event and may have caused the subsequent increase in population size. Our results indicate that applying CSIA can reveal strata-specific feeding grounds across the water column and can concurrently provide information about the nutritional quality of strata-specific resources for zooplankton.

The compound-specific dual-isotope approach of LIN and ALA allowed for a clear isotopic differentiation between diet sources along the lake water column of the stratified lake over the 6-week study period. Spatial and temporal changes of their isotopic values did not correlate, which would be the case if temperature had been the main driver. This indicates that differences are probably mainly driven by changes in the seston’s taxonomic composition (Zhang et al. 2009; Zhang and Sachs 2007). A precipitation event in the middle of the sampling period altered the bulk stable isotope values of the resources, thereby eliminating the 5 ‰ difference in δ^15^N between hypolimnion and the upper layers of the stratified lake, which was the only observed difference in bulk stable isotopes (Pilecky et al. 2023a). Extreme precipitation events and flooding typically export large amounts of allochthonous organic matter to lakes, which for oligotrophic lakes can lead to short-term severe eutrophication effects and may cause epilimnetic cyanobacterial blooms (Calderó-Pascual et al. 2020; Kasprzak et al. 2017; McCullough et al. 2012). In this case, the precipitation event probably triggered the decline of crysophyte biomass, thereby pushing PUFA, especially DHA-rich detritus, into deeper lake layers which eventually provides zooplankton species that forage at deeper lake layers with high-quality diet.

The increase in δ^2^H_LIN_ and δ^2^H_DHA_ values of hypolimnic seston may have been due to a combination of increased δ^2^H values of the ambient water, increase of water temperature in the metalimnion by the precipitation event, and a shift in dominant algal species, all of which can alter the isotopic fractionation of deuterium during FA synthesis (Zhang and Sachs 2007); however, their isotopic values remain “locked” once the FA have been formed (Sessions et al. 1999). Subsequently, the newly formed FA were passed on to zooplankton, which modify the FA profiles according to their specific physiological needs, which may be temperature sensitive (Gladyshev et al. 2011; McMeans et al. 2015). Saturated and monounsaturated FA are catabolized for gaining energy, thereby significantly altering their isotopic values, *i.e.*, isotopic enrichment relative to seston, and thus rendering them less useful for tracking dietary sources in zooplankton (Pilecky et al. 2022c, 2022b). In contrast, PUFA are highly retained by consumers, which makes them ideal candidates for food source tracking using CSIA (Koussoroplis et al. 2013). However, long-chain PUFA, such as ARA, EPA, and DHA, can be converted from their short-chain precursors (LIN and ALA), depending on the respective zooplankton physiology (Mathieu et al. 2022). This also significantly alters their isotopic values, particularly δ^2^H, which usually becomes more negative during conversion (Pilecky et al. 2022b). Indeed, we observed a striking difference in the range of diet and consumer δ^2^H_EPA_ values, *i.e.*, more negative in consumers, even after accounting for trophic isotope fractionation, suggesting that at least part of the EPA was converted from ALA by zooplankton. This renders LIN and ALA, which are found in high quantities in almost all food sources (Twining et al. 2016), as the most promising candidates for discerning feeding grounds of zooplankton, and possibly other aquatic consumers, without the need of accounting for metabolic processes.

The FA profiles of all zooplankton groups clearly differed from each other and their respective potential diet sources. The limitation of natural n-3 LC-PUFA resources in freshwater ecosystems led to a diversification of ecological strategies among zooplankton species. While cladocerans have a rapid, parthenogenic embryonal development and rely mainly on EPA to build up their neurological structures (Pilecky et al. 2022a), copepods feed selectively, using DHA as their main neuronal PUFA and also possess a more complex, obligatory sexual life cycle of various nauplius and copepodite stages before reaching their reproductive adult stages (Seebens et al. 2009; Titocci and Fink 2022). In experiments, the FA turnover rate of cladocerans was found to be approximately 5 days for ALA, and 6–7 days for LIN (Taipale et al. 2015, 2011), while it might take twice as long for copepods (Koussoroplis et al. 2014). These findings, however, are based on experiments with higher water temperature and thus faster metabolic rates (Gillooly et al. 2001; Ikeda 1985), which could explain the longer period of isotopic FA stability in the consumers of our lake study. We found the FA isotopic composition of zooplankton to remain similar up to two weeks after the precipitation event, despite shifts in isotopic composition of their food sources. Source isotope data should thus be collected over longer periods of time due to potential large variation caused by environmental influences, or taxonomic changes.

According to the Bayesian isotope mixing model, cladocerans had higher diet contribution of seston from upper lake strata, with a slightly higher preference for epilimnetic seston for *Daphnia*. Notably, the precipitation event coincided with a steep decline in the cladoceran population. Cladocerans are quickly reproducing and unselective filter-feeders, which is a rather cost-intensive strategy, especially during low dietary energy (Koenings et al. 1990). This might have contributed to the sudden population decline after the large precipitation event, which may have spilled high amounts of terrestrial organic matter with low dietary quality into the upper lake layers. The vertical movement of cladocerans is mainly regulated by visible light (420–600 nm), which indicates abundant food sources and induces positive phototaxis. However, the presence of predators, such as planktivorous fish, or UV-light (< 380 nm), can lead to vertical migration into deeper layers (Ringelberg 1999; Storz and Paul 1998). Accordingly, the model suggested that cladocerans utilized mainly resources from the upper lake layer. It is possible that the upward migrations of zooplankton to the epilimnion, in our case mostly cladocerans, increases the access to essential dietary FA, while it decreases predation risks during night (Ringelberg 2009). Bulk zooplankton biomass measured during the day and night in the summer of 2018 (see S6 to S8) was ~ 3 times higher in the epi- than meta- or hypolimnion, suggesting epilimnetic dietary provision of essential FA for cladocerans during the night.

The FA profiles of cyclopoids resembled those of calanoids, suggesting similar physiological requirements of n-3 PUFA; however, cyclopoids were preferentially feeding at epi- to metalimnetic layers. The higher light conditions for selective predation and exploitation of higher total biomass in the epi- and metalimnion fits the ecological strategy of cyclopoids, which share the feeding ground with cladocerans, indicating that diet acquisition from the epi- and metalimnion is generally more advantageous for obtaining large quantities of dietary energy. Cyclopoids have higher food threshold concentrations than calanoids (Santer 1994), which requires them to feed at lake layers with higher diet biomass. In contrast, calanoid copepods were mainly foraging in the meta- and hypolimnion. The increasing DHA content in the hypolimnion induced by the extreme precipitation event and the subsequent sinking of algal debris enhances the dietary access of DHA-linked neural development and reproduction of calanoids (Pilecky et al. 2021). Calanoid copepods are frequently associated with hypolimnetic habitats, where they selectively feed on DHA-rich diets, while sparing dietary sources associated with low food quality (*e.g.*, Balseiro et al. 2001; Kasprzak et al. 2005; Saage et al. 2009). This lake strata-specific feeding link with dietary DHA was corroborated by a 2 to threefold increase in calanoid copepod biomass after the precipitation event, while the biomass of hypolimnetic seston did not change significantly. Those were the only zooplankton groups, whose population did not decline because of the precipitation event. It might thus be speculated that calanoids require specific niches in lakes, where low temperature linked with lower metabolic rates support more slowly developing organisms, which are able to selectively feed on particles with high dietary quality. While both calanoids and cyclopoids are selective feeders, they have different ecological strategies and life-cycle histories (Adrian 1997). Cyclopoids are associated with higher carnivory (Maier 1990; Papirńska 1985) compared to calanoids. Furthermore, higher cyanobacterial consumption compared to calanoids indicate less selective feeding strategies in cyclopoids (Adrian 1997). Generally, calanoids show more stable population numbers throughout the year, while cyclopoids can have multiple seasonal population peaks (Adrian 1997). The ratio of calanoids to cyclopoids tends to decline with eutrophication (Maier 1996), with an increase in available dietary energy and a simultaneous decline in dietary quality (Taipale et al. 2016). This is suggested by our study, as the increase in selectively available DHA-rich particles coincided with higher calanoid population, while the decrease in available dietary energy per unit biomass may have led to a decline in the cyclopoid population.

## Conclusion

The presented dual-isotope (δ^13^C and δ^2^H) CSIA of FA approach for discerning feeding grounds of primary consumers provides an alternative to bulk stable isotope analyses, particularly in cases where the potential dietary resources exhibit low isotopic differentiation of bulk C or H. In contrast to studies monitoring only the presence of zooplankton at a certain depth, CSIA of FA provides long-term information on the preferential feeding grounds and is robust to time-dependent environmental fluctuations and extreme events. Furthermore, the observed preference of calanoids for hypolimnetic dietary sources in this specific case could help explain their population dynamics after an extreme precipitation event, which led to an increase in high quality hypolimnetic food sources. The identification of zooplankton feeding grounds within lake strata using CSIA also comes along with the provision of dietary quality data because dietary FA mass fractions at specific lake layers are concurrently identified and can thus be used as a measure for dietary energy acquisition. In this study, the decreasing δ^2^H_FA_ values indicate FA conversion and thus a potential mismatch between diet and physiological requirements of zooplankton.

## Supplementary Information

Below is the link to the electronic supplementary material.

Supplementary file1 (PDF 1168 kb)

## Data Availability

Data can be requested directly from the corresponding author.
