# Supplementary material for: Compound-specific stable isotope analyses of fatty acids indicate feeding zones of zooplankton across the water column of a subalpine lake
Source: Oecologia. 2024 Jun 3;205(2):325–37. doi: 10.1007/s00442-024-05574-3 (PMC11628586; doi:10.1007/s00442-024-05574-3)

# Compound-specific stable isotope analyses of fatty acids indicate feeding zones of zooplankton across the water column of a subalpine lake

Supplementary Material

Matthias Pilecky[1,2], Samuel K. Kämmer[1], Katharina Winter[1], Radka Ptacnikova[1], Travis B. Meador[3,4], Leonard I. Wassenaar[1], Patrick Fink[5,6] and Martin J. Kainz[1,2]

1) WasserCluster Lunz – Biologische Station, Inter-University Center for Aquatic Ecosystem Research, Dr. Carl-Kupelwieser Promenade 5, 3293 Lunz/See, Austria
2) Donau-Universität Krems, Research Lab for Aquatic Ecosystem Research and -Health, Dr. Karl-Dorrek Straße 30, 3500 Krems, Austria
3) University of Southern Bohemia, Na Sádkách 7, 370 05 České Budějovice, Czech Republic
4) Biology Center CAS, Na Sádkách 7, 370 05 České Budějovice, Czech Republic
5) Helmholtz Centre for Environmental Research – UFZ, Department River Ecology, Brückstraße 3a, 39114 Magdeburg, Germany
6) Helmholtz Centre for Environmental Research – UFZ, Department Aquatic Ecosystem Analysis and Management, Brückstraße 3a, 39114 Magdeburg, Germany

E-mail:

MP: matthias.pilecky@donau-uni.ac.at

MJK: martin.kainz@donau-uni.ac.at

PF: patrick.fink@ufz.de

Running Headline: CSIA discern zooplankton foraging depth

MP and MJK conceived the ideas and designed methodology; MP, KW and SK conducted field work; MP, KW, RP and SK collected and analyzed the data; MP and PF performed statistical analysis and led the writing of the manuscript. TBM, LIW and MJK provided editorial supervision and critically revised the manuscript.

**Table S1. Phytoplankton from Lake Lunz around the flood event in July 2021.** Samples were mixed with Lugol and transfered to Uttermöhl chamber of Volume 25 mL, sedimented overnight before examination.

| Date | Layer | Depth [m] | Dominant species |
|---|---|---|---|
| **22.6. (Secchi 6,4 m)** | **Epi** | **5** | *Dinobryon* (chrysophyts/Dinobryaceae) - by far most dominant<br>Further present: *Gymnodinium sp.* (dinoflagellates) |
| | **Meta** | **10** | *Dinobryon* (chrysophyts/Dinobryaceae) - by far most dominant<br>Further present: *Cyclotella*, *Asterionella* (diatoms)<br>*Gymnodinium helveticum* (dinoflagellates) |
| | **Hypo** | **25** | Significantly less particles, mainly detritus<br>A bit of *Dinobryon* and diatoms |
| **20.7. (Secchi 1,8 m)** | **Epi** | **3** | No clear dominance, seems a lot of detritus, which might be also disassembled colonies of *Uroglena* (chrysophytes)<br>*Dinobryon* (way less than a month ago)<br>*Ceratium* (dinoflagellates) (little)<br>Diatoms (little) |
| | **Meta** | **12** | A lot of 'debris' (see above)<br>a bit of *Dinobryon* |
| | **Hypo** | **25** | A lot of 'debris' (see above)<br>a bit of *Dinobryon*<br>some diatoms |
| **17.8.<br><br>(Secchi 8,8 m)** | **Epi** | **3** | Not really one dominant group<br>Present: *Dinobryon* & *Gymnodinium helveticum* (dinoflagellates), *Asterionella* (diatoms), *Mallomonas* (chrysophytes), *Staurastrum* (desmidids), *Coleps* (ciliate) |
| | **Meta** | **12** | Not really one dominant group<br>Present: *Dinobryon* & *Gymnodinium helveticum* & *Ceratium* (dinoflagellates), *Asterionella* (diatoms), *Mallomonas* (chrysophytes), *Cryptomonas* spp.(cryptophytes), *Coleps* (ciliate) |
| | **Hypo** | **25** | Practically only detritus |

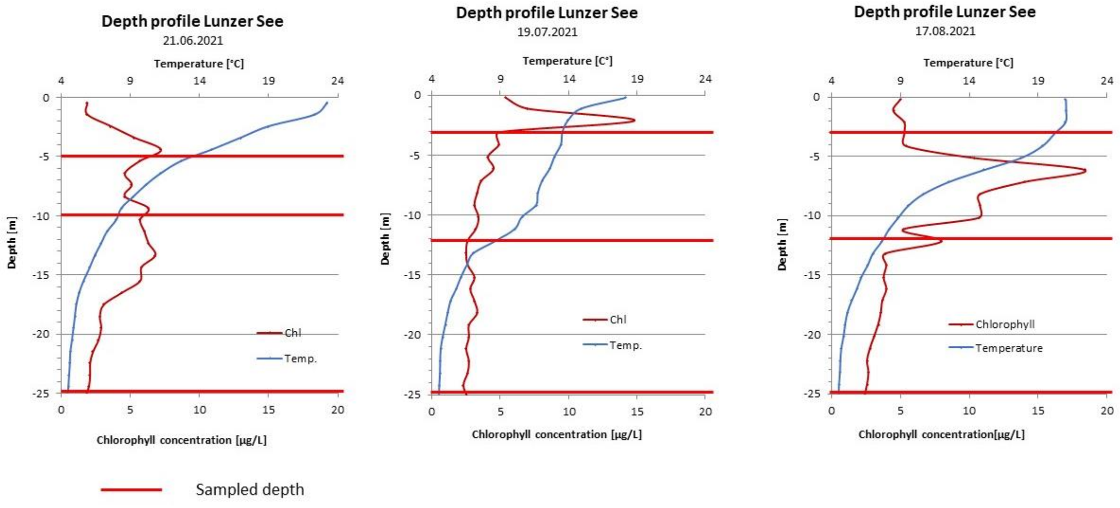

**Table S2. Average mass fractions of zooplankton species and seston of different lake layers. Values are given in mg/g dry weight (± sd).**

| | Bosmina | Calanoid | Cyclops | Daphnia | Epilimnion | Metalimnion | Hypolimnion |
|---|---|---|---|---|---|---|---|
| **14:0** | 16.0 (6.6) | 6.0 (3.9) | 14.3 (5.9) | 6.8 (3.3) | 1.1 (0.5) | 1.4 (0.8) | 1.5 (0.9) |
| **14:1n-5** | 0.3 (0.2) | 0.1 (0.0) | 0.2 (0.1) | 0.5 (0.4) | 0.0 (0.1) | 0.1 (0.1) | 0.0 (0.0) |
| **15:0** | 0.8 (0.4) | 0.5 (0.2) | 1.0 (0.4) | 1.2 (0.6) | 0.3 (0.2) | 0.3 (0.3) | 0.4 (0.2) |
| **15:1n-5** | 0.1 (0.1) | 0.1 (0.1) | 0.0 (0.0) | 0.1 (0.1) | 0.0 (0.0) | 0.0 (0.1) | 0.0 (0.0) |
| **16:0** | 22.4 (10.5) | 18.1 (11.4) | 25.8 (9.7) | 16.3 (6.5) | 3.6 (1.3) | 3.8 (1.7) | 4.8 (1.9) |
| **16:1n-7** | 5.3 (3.0) | 1.8 (0.6) | 3.9 (1.9) | 6.5 (4.2) | 0.6 (0.2) | 0.8 (0.5) | 0.7 (0.4) |
| **16:1n-9** | 3.6 (4.4) | 1.4 (2.0) | 1.7 (2.9) | 2.8 (5.0) | 0.6 (0.6) | 0.7 (0.6) | 0.8 (0.4) |
| **17:0** | 0.4 (0.3) | 0.5 (0.3) | 0.5 (0.2) | 0.5 (0.3) | 0.1 (0.0) | 0.1 (0.1) | 0.1 (0.1) |
| **17:1n-7** | 0.3 (0.5) | 0.3 (0.4) | 0.3 (0.3) | 0.5 (0.7) | 0.1 (0.1) | 0.1 (0.1) | 0.1 (0.1) |
| **18:0** | 4.4 (4.0) | 6.1 (4.5) | 5.1 (2.2) | 4.7 (3.6) | 1.6 (0.7) | 1.5 (0.8) | 2.0 (1.0) |
| **18:1n-12** | 0.2 (0.3) | 0.1 (0.1) | 0.1 (0.1) | 0.2 (0.3) | 0.1 (0.2) | 0.2 (0.2) | 0.2 (0.1) |
| **18:1n-6** | 0.0 (0.0) | 0.1 (0.1) | 0.1 (0.1) | 0.1 (0.2) | 0.1 (0.1) | 0.1 (0.1) | 0.1 (0.2) |
| **18:1n-7** | 4.0 (2.1) | 1.5 (0.9) | 2.1 (0.8) | 3.7 (1.5) | 0.3 (0.1) | 0.2 (0.1) | 0.3 (0.2) |
| **18:1n-9** | 16.6 (7.7) | 6.2 (3.0) | 13.5 (6.1) | 8.2 (3.8) | 1.0 (0.5) | 1.4 (0.7) | 1.8 (1.1) |
| **18:2n-6** | 11.1 (8.7) | 5.6 (5.5) | 10.3 (7.9) | 6.2 (4.8) | 0.7 (0.3) | 1.0 (0.6) | 1.5 (1.0) |
| **18:3n-3** | 4.7 (2.7) | 4.1 (2.8) | 5.1 (1.9) | 3.8 (2.0) | 0.7 (0.4) | 0.6 (0.6) | 0.7 (0.6) |
| **18:3n-6** | 0.7 (0.4) | 0.3 (0.3) | 0.5 (0.3) | 0.6 (0.4) | 0.1 (0.0) | 0.1 (0.0) | 0.1 (0.0) |
| **18:4n-3** | 19.6 (10.4) | 6.2 (3.9) | 11.6 (4.7) | 7.6 (3.8) | 0.9 (0.4) | 1.0 (0.8) | 1.0 (0.6) |
| **20:0** | 0.2 (0.2) | 0.3 (0.2) | 0.5 (0.2) | 0.1 (0.1) | 0.1 (0.0) | 0.1 (0.0) | 0.1 (0.1) |
| **20:1n-9** | 0.2 (0.7) | 0.4 (0.8) | 0.8 (1.3) | 0.2 (0.7) | 0.1 (0.2) | 0.1 (0.2) | 0.0 (0.2) |
| **20:2n-6** | 0.1 (0.1) | 0.6 (0.4) | 0.5 (0.2) | 0.1 (0.1) | 0.0 (0.0) | 0.0 (0.0) | 0.0 (0.0) |
| **20:3n-3** | 0.6 (1.0) | 0.5 (0.8) | 0.5 (0.6) | 1.1 (2.0) | 0.0 (0.0) | 0.0 (0.1) | 0.0 (0.1) |
| **20:3n-6** | 1.4 (2.1) | 0.2 (0.1) | 0.6 (0.7) | 0.6 (1.2) | 0.6 (0.3) | 0.0 (0.1) | 0.1 (0.2) |
| **20:4n-3** | 0.5 (0.3) | 0.6 (0.4) | 2.4 (1.2) | 0.4 (0.2) | 0.1 (0.1) | 0.1 (0.1) | 0.1 (0.1) |
| **20:4n-6** | 3.9 (3.0) | 3.4 (1.8) | 2.4 (1.2) | 4.1 (2.6) | 0.3 (0.2) | 0.2 (0.2) | 0.3 (0.2) |
| **20:5n-3** | 14.2 (12.1) | 9.5 (7.2) | 7.4 (4.6) | 9.4 (7.0) | 0.7 (0.3) | 0.7 (0.4) | 0.9 (0.5) |
| **22:0** | 1.6 (2.9) | 0.4 (0.4) | 0.6 (0.8) | 0.5 (0.8) | 0.1 (0.0) | 0.1 (0.1) | 0.1 (0.1) |
| **22:1n-9** | 0.1 (0.1) | 0.1 (0.1) | 0.4 (0.2) | 0.0 (0.0) | 0.0 (0.1) | 0.0 (0.0) | 0.0 (0.0) |
| **22:2n-6** | 0.0 (0.0) | 0.0 (0.1) | 0.1 (0.0) | 0.0 (0.0) | 0.0 (0.1) | 0.0 (0.1) | 0.0 (0.1) |
| **22:3n-3** | 0.1 (0.3) | 0.7 (1.0) | 0.5 (0.9) | 0.1 (0.1) | 0.0 (0.0) | 0.0 (0.1) | 0.0 (0.1) |
| **22:4n-6** | 0.2 (0.4) | 1.5 (2.5) | 0.7 (1.1) | 0.1 (0.2) | 0.0 (0.0) | 0.0 (0.0) | 0.0 (0.0) |
| **22:5n-3** | 0.2 (0.1) | 0.4 (0.3) | 2.2 (1.0) | 0.2 (0.1) | 0.0 (0.0) | 0.0 (0.0) | 0.0 (0.0) |
| **22:6n-3** | 6.9 (3.1) | 21.4 (12.9) | 21.6 (8.2) | 2.2 (1.5) | 0.9 (0.3) | 1.2 (0.6) | 2.0 (1.1) |
| **24:0** | 0.1 (0.1) | 0.1 (0.0) | 0.1 (0.1) | 0.1 (0.1) | 0.1 (0.0) | 0.1 (0.1) | 0.1 (0.1) |
| **24:1n-9** | 2.9 (5.4) | 1.6 (1.7) | 2.1 (2.7) | 1.8 (3.7) | 0.0 (0.0) | 0.0 (0.0) | 0.1 (0.2) |
| **SFA** | 45.9 (18.6) | 32.2 (19.7) | 48.0 (17.5) | 30.3 (12.0) | 6.9 (2.4) | 7.9 (3.5) | 9.4 (3.7) |
| **MUFA** | 33.6 (14.0) | 13.7 (6.8) | 25.2 (11.2) | 24.6 (8.9) | 3.0 (1.2) | 3.9 (1.8) | 4.2 (1.7) |
| **PUFA** | 64.9 (34.9) | 55.1 (37.0) | 66.5 (25.3) | 36.9 (16.9) | 5.2 (1.8) | 5.6 (2.9) | 6.8 (3.4) |
| **n-3 PUFA** | 46.9 (27.3) | 43.4 (27.5) | 51.2 (19.1) | 24.8 (12.0) | 3.3 (1.2) | 4.0 (2.4) | 4.7 (2.4) |
| **n-6 PUFA** | 18.0 (11.0) | 11.8 (10.1) | 15.3 (8.9) | 12.1 (6.6) | 2.0 (0.7) | 1.7 (0.8) | 2.2 (1.2) |
| **BFA** | 13.1 (9.1) | 6.3 (3.3) | 9.9 (7.2) | 10.2 (4.9) | 1.4 (0.6) | 1.4 (0.9) | 1.6 (0.6) |
| **TFA** | 1.8 (2.9) | 0.8 (0.5) | 1.2 (1.0) | 0.7 (0.8) | 0.2 (0.1) | 0.3 (0.1) | 0.4 (0.2) |
| **n-3/n-6** | 3.6 (3.3) | 4.8 (2.5) | 4.8 (3.8) | 2.3 (1.3) | 1.8 (0.7) | 2.7 (1.6) | 2.3 (1.5) |
| **Total FAME** | 152.6 (63.4) | 104.8 (63.6) | 146.0 (54.9) | 96.8 (36.7) | 15.7 (5.1) | 17.9 (7.9) | 21.1 (8.5) |

**Figure S3. Changes in FA parameters of seston in all three layers.** Mass fraction (black line); relative mass fraction compared to total FAME (gray area); δ$^{2}$H (blue, in % vs. VSMOW); and δ$^{13}$C (red in ‰ vs. VPDB) following the precipitation event (blue vertical line).

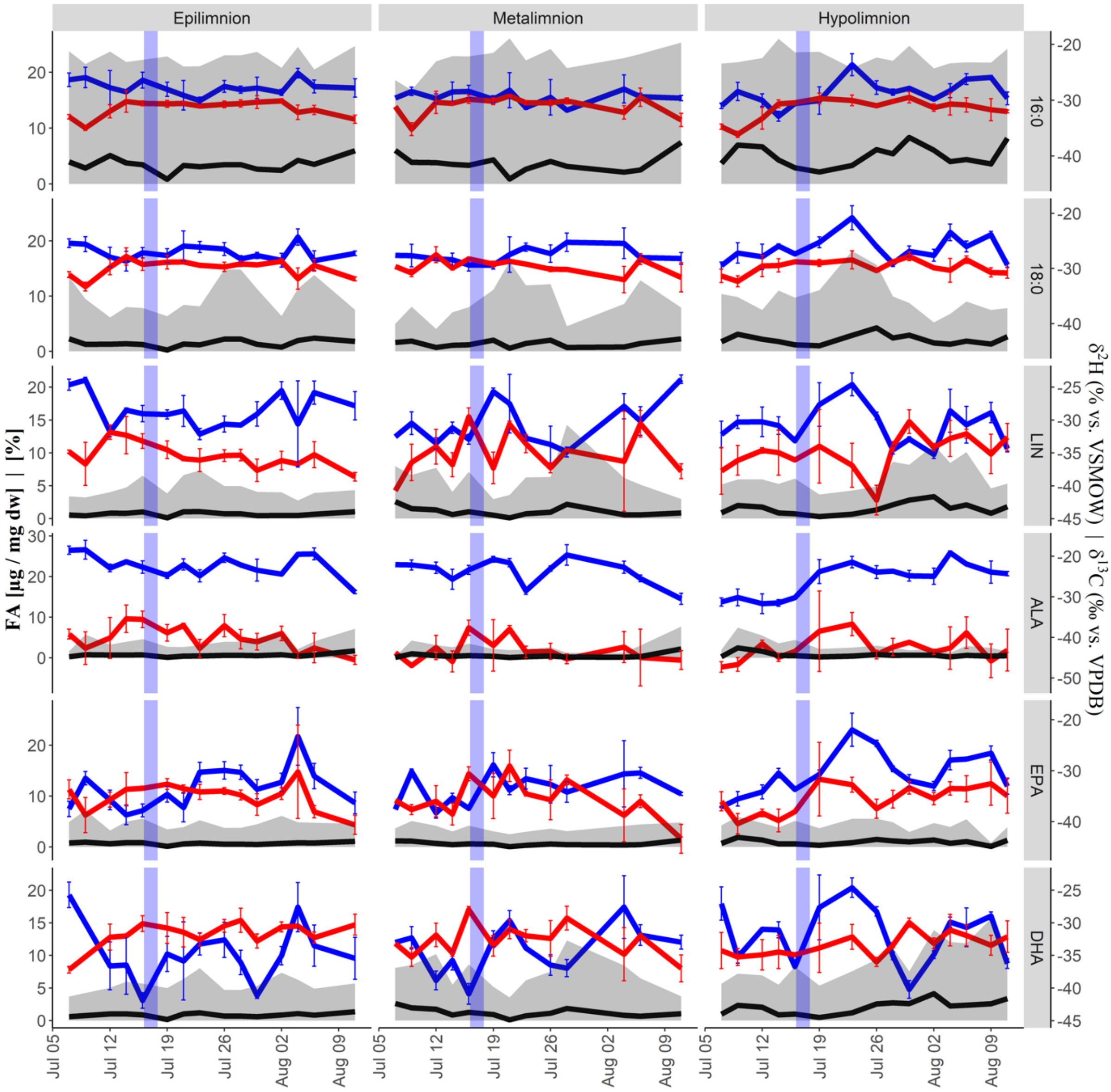

**Table S4. Mean and Standard deviation of mass fraction, relative mass fraction compared to total FA, $\delta^2$H and $\delta^{13}$C values before (7th to 14th of July), immediately after (19th to 23rd of July) and in a late phase (July 28 to August 6) after the flooding event.**

| | | $\delta^2$H [vs. VSMOW] | | | $\delta^{13}$C [vs. VPDB] | | | mass fraction [µg/mg dw] | | | relative abundance [FAME mass %] | | |
|---|---|---|---|---|---|---|---|---|---|---|---|---|---|
| | | **Epi** | **Met** | **Hyp** | **Epi** | **Met** | **Hyp** | **Epi** | **Met** | **Hyp** | **Epi** | **Met** | **Hyp** |
| **16:0** | *before Event* | -269.91 (10.91) | -289.37 (6.68) | -305.87 (16.14) | -32.14 (1.92) | -31.41 (2.16) | -33.04 (2.49) | 3.83 (0.83) | 4.12 (1.09) | 4.90 (1.82) | 22.61 (1.07) | 20.62 (2.77) | 23.09 (1.80) |
| | *after Event* | -290.82 (10.19) | -297.93 (15.61) | -268.52 (45.37) | -30.75 (0.25) | -29.96 (0.65) | -29.86 (0.25) | 2.42 (1.38) | 2.63 (1.71) | 2.73 (0.82) | 21.66 (1.03) | 23.89 (2.01) | 22.22 (2.07) |
| | *late phase* | -273.57 (15.31) | -297.18 (19.24) | -282.25 (14.86) | -31.16 (0.96) | -30.60 (1.41) | -30.77 (0.47) | 3.42 (0.73) | 2.60 (0.53) | 4.97 (0.93) | 22.23 (1.91) | 22.20 (1.30) | 21.54 (0.59) |
| **18:0** | *before Event* | -269.45 (14.32) | -282.56 (7.08) | -275.64 (12.88) | -30.25 (2.07) | -29.22 (1.33) | -30.29 (1.49) | 1.49 (0.45) | 1.29 (0.45) | 2.00 (0.71) | 9.00 (2.76) | 6.44 (1.83) | 9.69 (1.34) |
| | *after Event* | -265.68 (9.44) | -276.70 (16.35) | -230.31 (31.45) | -29.03 (0.35) | -29.03 (0.29) | -28.71 (0.39) | 0.92 (0.59) | 1.34 (0.75) | 1.96 (1.35) | 7.65 (1.11) | 13.11 (2.73) | 14.62 (5.02) |
| | *late phase* | -274.14 (20.79) | -262.31 (15.40) | -265.39 (24.05) | -29.78 (1.40) | -30.16 (1.86) | -29.35 (0.90) | 1.83 (0.74) | 0.97 (0.39) | 1.77 (0.49) | 11.60 (3.76) | 8.68 (4.16) | 7.75 (2.13) |
| **LIN 18:2n-6** | *before Event* | -275.74 (32.75) | -321.30 (12.67) | -313.52 (12.74) | -33.77 (1.98) | -35.51 (4.13) | -35.90 (1.22) | 0.73 (0.23) | 1.43 (0.74) | 1.25 (0.58) | 4.39 (1.34) | 6.72 (1.57) | 5.71 (0.43) |
| | *after Event* | -299.69 (18.97) | -286.42 (36.71) | -261.15 (21.67) | -35.50 (0.85) | -33.85 (3.33) | -35.44 (2.05) | 0.76 (0.54) | 0.47 (0.32) | 0.50 (0.25) | 5.94 (1.85) | 4.28 (1.76) | 3.96 (0.42) |
| | *late phase* | -281.73 (28.93) | -309.69 (35.58) | -322.74 (31.59) | -35.87 (0.68) | -33.81 (2.94) | -33.27 (0.99) | 0.59 (0.14) | 1.11 (0.93) | 2.28 (0.75) | 3.91 (0.90) | 8.55 (4.99) | 9.62 (1.29) |
| **ALA** | *before Event* | -207.39 (21.89) | -231.94 (14.74) | -309.39 (7.46) | -38.57 (3.11) | -43.33 (3.64) | -44.76 (2.34) | 0.62 (0.19) | 0.50 (0.33) | 1.09 (0.92) | 3.83 (1.54) | 2.75 (1.53) | 4.56 (2.05) |
| **18:3n-3** | *after Event* | -238.34 (15.93) | -235.95 (42.53) | -226.02 (16.57) | -39.53 (2.87) | -41.16 (2.87) | -37.57 (1.24) | 0.33 (0.20) | 0.23 (0.17) | 0.30 (0.14) | 2.85 (0.27) | 2.06 (0.13) | 2.36 (0.24) |
| | *late phase* | -213.48 (23.90) | -226.01 (28.88) | -224.00 (25.05) | -41.61 (2.39) | -44.19 (1.59) | -41.83 (2.07) | 0.60 (0.16) | 0.20 (0.08) | 0.52 (0.19) | 4.13 (1.73) | 1.84 (1.02) | 2.22 (0.71) |
| **EPA** | *before Event* | -362.53 (28.70) | -357.60 (33.00) | -341.98 (24.76) | -35.04 (2.25) | -35.73 (3.08) | -38.52 (1.71) | 0.83 (0.11) | 0.84 (0.33) | 1.05 (0.57) | 5.19 (1.43) | 4.05 (0.75) | 4.78 (0.83) |
| **20:5n-3** | *after Event* | -340.62 (35.16) | -314.16 (24.97) | -263.43 (62.01) | -33.44 (0.77) | -32.85 (3.29) | -32.22 (0.78) | 0.51 (0.34) | 0.33 (0.23) | 0.61 (0.37) | 4.23 (0.94) | 2.86 (0.31) | 4.62 (1.30) |
| | *late phase* | -292.34 (40.50) | -317.50 (21.29) | -295.79 (24.54) | -34.39 (3.22) | -35.54 (3.52) | -34.54 (1.17) | 0.74 (0.12) | 0.44 (0.04) | 1.10 (0.28) | 4.89 (0.97) | 3.91 (0.70) | 4.75 (0.61) |
| **DHA** | *before Event* | -351.79 (68.08) | -361.54 (37.05) | -322.09 (38.53) | -32.88 (3.03) | -32.57 (2.90) | -34.75 (0.39) | 0.88 (0.19) | 1.70 (0.68) | 1.45 (0.69) | 5.10 (1.00) | 8.19 (1.70) | 6.73 (1.15) |
| **22:6n-3** | *after Event* | -346.21 (13.13) | -319.78 (21.52) | -261.06 (21.45) | -31.60 (0.91) | -32.16 (1.28) | -32.99 (1.20) | 0.80 (0.54) | 0.60 (0.42) | 0.84 (0.50) | 6.48 (1.54) | 4.98 (1.34) | 6.47 (1.55) |
| | *late phase* | -330.50 (38.00) | -321.31 (46.87) | -326.28 (27.27) | -30.74 (1.11) | -32.02 (2.77) | -32.40 (1.08) | 0.87 (0.16) | 1.11 (0.66) | 2.87 (0.87) | 5.81 (1.25) | 9.09 (2.95) | 12.25 (1.16) |

**Table S5. Mean and Standard deviation of mean global δ$^2$H and δ$^{13}$C values of zooplankton genera.**

| | δ$^2$H values | | | | δ$^{13}$C values | | | |
|---|---|---|---|---|---|---|---|---|
| | **Bosmina** | **Calanoids** | **Cyclopoids** | **Daphnia** | **Bosmina** | **Calanoids** | **Cyclopoids** | **Daphnia** |
| **14:0** | -315.5 ± 21.6 | -304.9 ± 28.6 | -306.3 ± 33.8 | -299.9 ± 37.1 | -33.8 ± 1.0 | -34.1 ± 1.3 | -34.0 ± 1.2 | -34.4 ± 1.4 |
| **15:0** | -274.2 ± 60.2 | -254.8 ± 78.5 | -295.5 ± 44.7 | -279.0 ± 62.0 | -35.4 ± 1.5 | -33.7 ± 1.9 | -37.8 ± 4.3 | -36.3 ± 1.5 |
| **16:0** | -302.6 ± 14.8 | -302.0 ± 22.4 | -298.6 ± 18.5 | -272.2 ± 20.4 | -32.6 ± 1.0 | -33.9 ± 0.6 | -32.1 ± 7.4 | -33.7 ± 1.5 |
| **16:1** | -346.9 ± 28.0 | -355.9 ± 56.5 | -348.6 ± 50.5 | -325.6 ± 33.8 | -34.6 ± 1.5 | -36.2 ± 2.0 | -35.5 ± 6.4 | -37.6 ± 6.6 |
| **18:0** | -251.5 ± 30.2 | -251.8 ± 26.6 | -229.6 ± 30.1 | -245.8 ± 49.2 | -31.5 ± 3.0 | -33.7 ± 1.1 | -34.0 ± 2.1 | -32.7 ± 2.8 |
| **18:1** | -286.3 ± 17.3 | -279.1 ± 20.2 | -286.8 ± 21.7 | -256.8 ± 44.0 | -33.2 ± 1.9 | -35.0 ± 1.2 | -33.5 ± 2.8 | -35.8 ± 2.2 |
| **20:1** | -372.0 ± 14.9 | -331.6 ± 26.3 | -279.5 ± 43.9 | -333.6 ± 68.9 | -34.5 ± 2.7 | -38.6 ± 2.5 | -34.8 ± 2.9 | -38.0 ± 3.6 |
| **LIN** | -330.5 ± 19.3 | -319.3 ± 24.2 | -322.4 ± 14.8 | -297.5 ± 27.5 | -34.1 ± 2.3 | -37.3 ± 1.8 | -34.3 ± 3.2 | -36.8 ± 3.0 |
| **GLA** | -253.6 ± 77.0 | -180.1 ± 190.3 | -281.2 ± 98.0 | -275.2 ± 76.6 | -39.2 ± 4.8 | -38.1 ± 5.3 | -37.9 ± 5.6 | -40.4 ± 4.2 |
| **ARA** | -269.0 ± 26.6 | -262.2 ± 32.8 | -249.5 ± 53.3 | -260.7 ± 35.2 | -39.0 ± 2.3 | -37.6 ± 2.3 | -37.3 ± 3.1 | -37.0 ± 2.5 |
| **AdA** | -295.7 ± NA | -224.6 ± 44.1 | -215.5 ± 29.2 | N/A | -39.7 ± NA | -40.9 ± 2.9 | -39.5 ± 3.7 | N/A |
| **ALA** | -219.9 ± 24.6 | -260.0 ± 26.3 | -245.3 ± 21.2 | -239.5 ± 28.2 | -42.2 ± 2.4 | -44.1 ± 2.0 | -42.2 ± 3.0 | -42.1 ± 3.1 |
| **SDA** | -356.8 ± 15.5 | -350.9 ± 33.2 | -369.9 ± 20.4 | -358.0 ± 34.7 | -35.5 ± 1.7 | -39.3 ± 3.0 | -35.5 ± 2.8 | -37.8 ± 3.1 |
| **ETA** | -194.8 ± 31.4 | N/A | -295.0 ± 55.7 | -222.4 ± 53.4 | -39.4 ± 8.7 | N/A | -36.7 ± 2.2 | -37.6 ± 3.1 |
| **EPA** | -370.3 ± 24.7 | -343.3 ± 37.6 | -357.2 ± 32.1 | -342.0 ± 38.7 | -33.5 ± 2.1 | -37.7 ± 2.4 | -37.0 ± 3.3 | -35.6 ± 2.5 |
| **DHA** | -317.9 ± 53.6 | -325.6 ± 30.8 | -338.8 ± 41.7 | -298.3 ± 77.7 | -32.3 ± 2.2 | -34.9 ± 1.8 | -33.0 ± 2.3 | -35.3 ± 2.7 |

**Figure S6** Day/night around midday/midnight on 16./17. 8. and 7./8. 9. 2018, again with closable 40 µm nets deployed according to actual temperature profiles

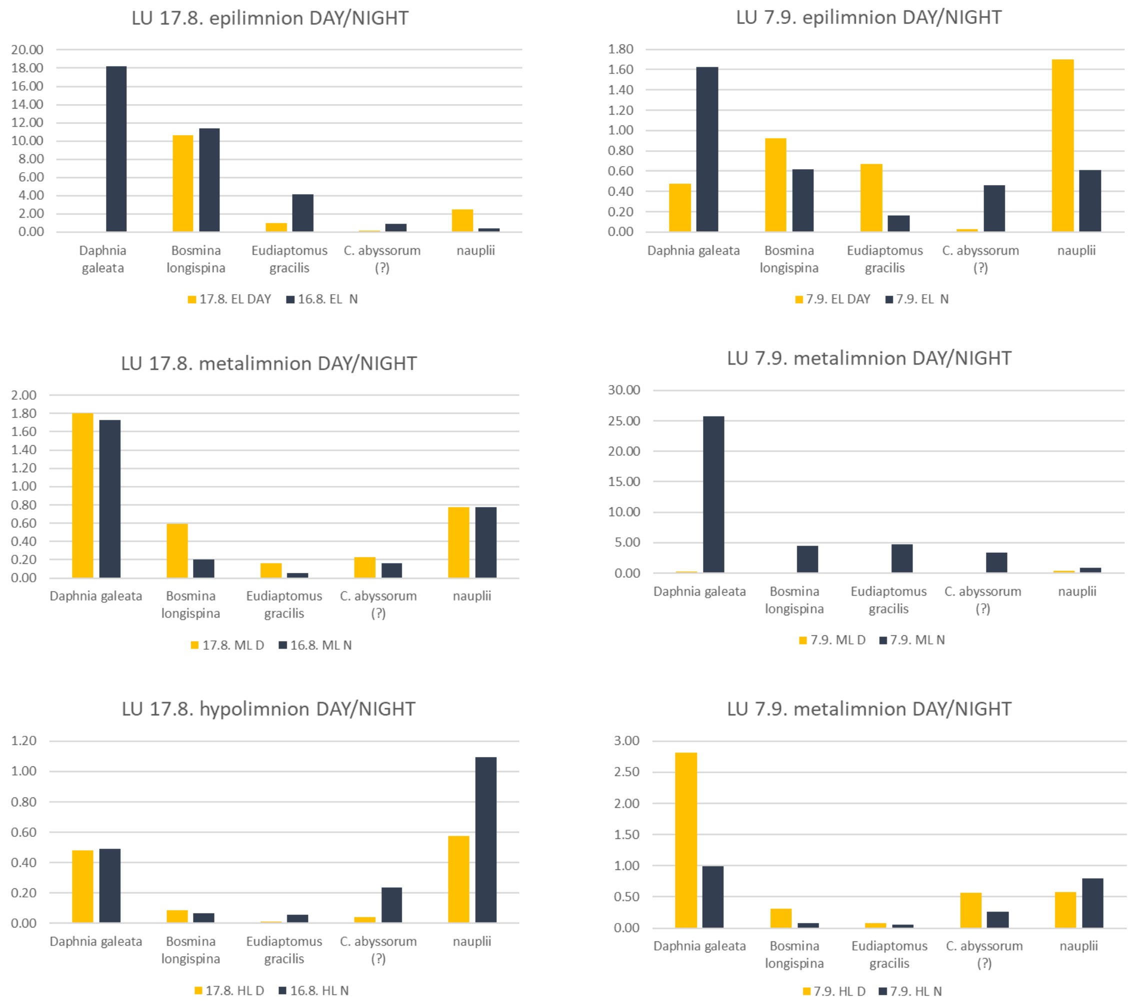

**Figure S7** Data from an underwater camera at the 16th and the night to 17th August 2018. Resolution at species level was not possible.

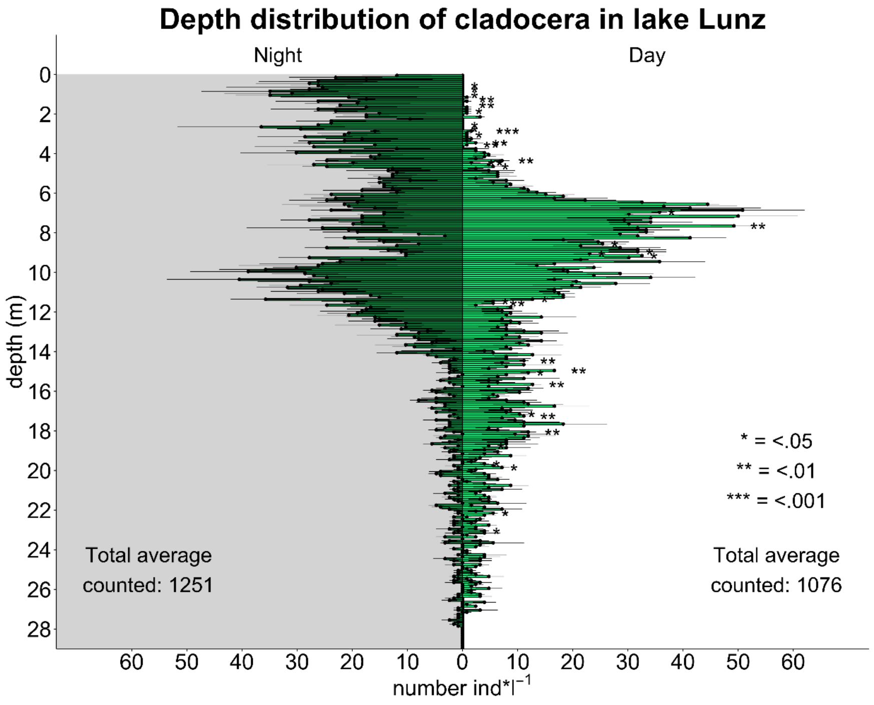


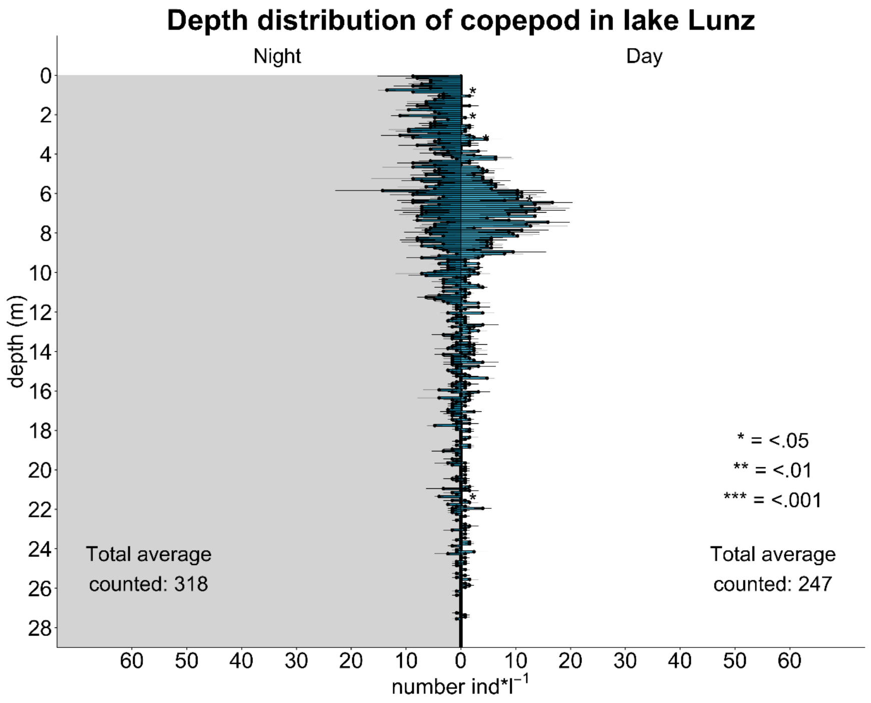

**Figure S8** Data from an underwater camera at the 7th and the night to 8th September 2018. Resolution at species level was not possible.

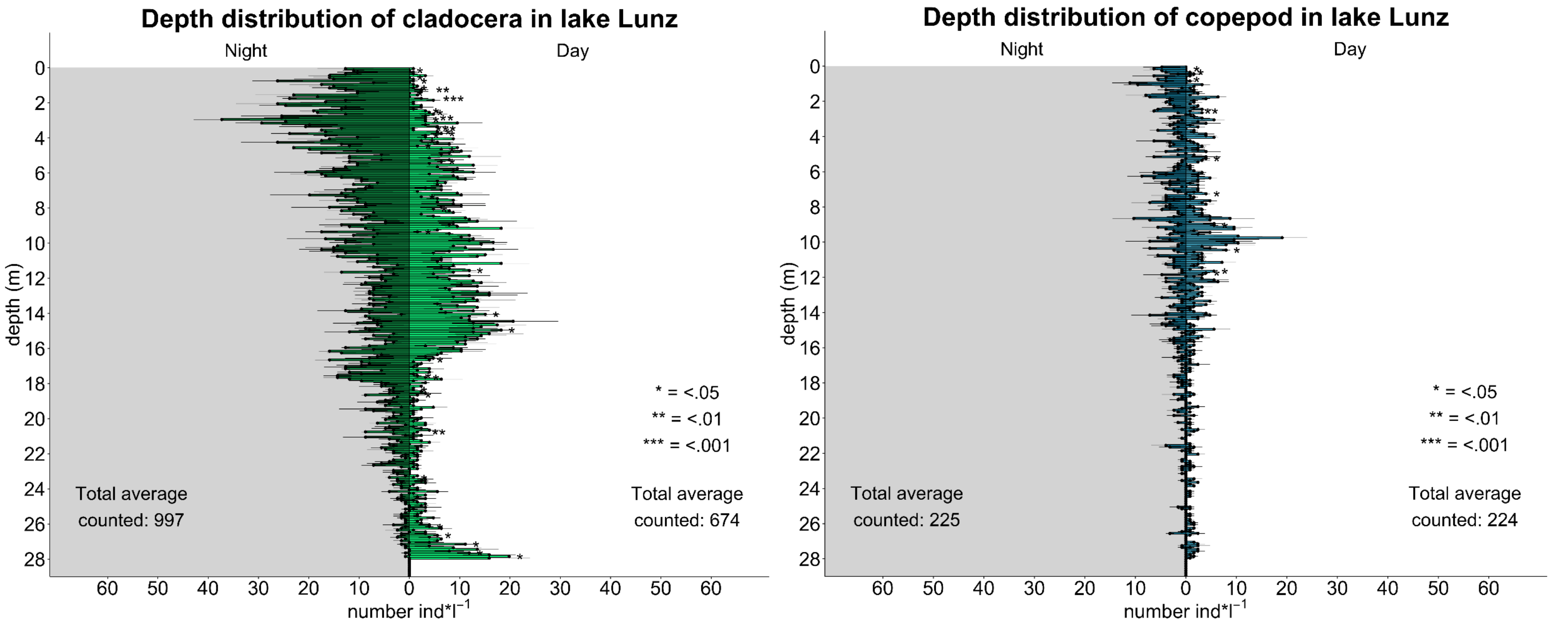

Supplement: Supplementary file 1 — Supplementary file1 (PDF 1168 kb) [file 442_2024_5574_MOESM1_ESM.pdf]
